# Employment, occupation, and income in adults with neurofibromatosis 1 in Denmark: a population- and register-based cohort study

**DOI:** 10.1186/s13023-023-02965-2

**Published:** 2023-11-06

**Authors:** Line Kenborg, Line E. Frederiksen, Michael Galanakis, Karoline Doser, Thomas T. Nielsen, Mia Aagaard Doherty, Hanne Hove, John R. Østergaard, Mette M. Handrup, Cecilie Ejerskov, John J. Mulvihill, Jeanette F. Winther

**Affiliations:** 1 Childhood Cancer Research Group, Danish Cancer Institute, Strandboulevarden 49, 2100 Copenhagen, Denmark; 2 Statistics and Data Analysis, Danish Cancer Institute, Copenhagen, Denmark; 3 Department of Pediatrics, Center for Rare Diseases, University Hospital at Rigshospitalet, Copenhagen, Denmark; 4 The RAREDIS Database, Center for Rare Diseases, Copenhagen University Hospital and Aarhus University Hospital, Copenhagen, Denmark; 5 Center for Rare Diseases, Aarhus University Hospital, Aarhus, Denmark; 6 Department of Pediatrics, University of Oklahoma, Oklahoma City, OK USA; 7 Department of Clinical Medicine, Faculty of Health, Aarhus University and University Hospital, Aarhus, Denmark

**Keywords:** Neurofibromatosis 1, Population-based, Cohort study, Employment status, Denmark

## Abstract

**Background:**

Little is known about employment status, occupation, and disposable income in adults with NF1.

**Methods:**

From the Danish National Patient Registry and database of two national Centers for Rare Diseases, we identified 1469 adults with NF1, who were matched to 11,991 randomly selected population comparisons on sex and birth year and month. Annual information on employment, occupation and disposable income was ascertained from national registries in 1980–2019.

**Results:**

Adults with NF1 had a lower odds ratio (OR) for employment [OR 0.71, 95% confidence interval (CI) 0.61–0.83] and higher OR for health-related unemployment (OR 2.94, 95% CI 2.16–3.96) at age 30 years than population comparisons, which persisted at age 40 and 50 years. Somatic diagnoses were associated with a higher OR for health-related unemployment in adults with NF1 than in the population comparisons. Adults with NF1 had a slightly lower disposable income, with a 14% (0.82–0.89) reduction observed among the youngest birth cohort. Furthermore, adults with NF1 were less likely to be in a high skilled occupation at ages 30, 40 and 50 years.

**Conclusion:**

Adults with NF1 have a lower employment rate, which was mainly due to health-related reasons and a slightly lower disposable income than adults without NF1. Thus, anticipation guidance for employment should be part of the management of NF1 families.

**Supplementary Information:**

The online version contains supplementary material available at 10.1186/s13023-023-02965-2.

## Introduction

Neurofibromatosis 1 (NF1 [MIM 162200]) is one of the most common single-gene disorders, with a reported birth incidence of up to 1/2000 [1]. Although NF1 can affect all organ systems of the body, cutaneous, neurologic, and orthopedic manifestations occur most often in individuals with NF1 [2]. NF1 has an extreme clinically variability, both within family members with the same variant, but also in an individual at different times in life [3]. Some individuals with NF1 will not be significantly affected on their health, while others experience severe psychiatric and medical morbidities [4, 5], including an increased risk of cancer [6], which may lead to a reduced life expectancy [1]. NF1 has a negative impact on quality of life [7, 8] and educational attainment [9, 10], which together with the high disease burden, may have long-term consequences for employment status, occupational position, and income.

A few cross-sectional studies have reported employment rates in adults with NF1 [7, 8, 11–13]. Based on self-reported data, employment rates have ranged between 41% in a Danish questionnaire study of 244 adults with NF1 [7] to 70% both (full- and part time) employment among 498 American adults with NF1 [11]. Recently, a Finnish study showed that individuals with NF1 had a lower number of working days per year, but they were more often unemployed and had more sickness absence than a control cohort [14]. Using the same study population, another Finnish study reported that NF1 decreased the probability of having a work-related income as well as the amount of annual earning among those having an income from work [15]. Due to the limited number of population-based studies evaluating work life of adults with NF1 in a population-based setting, we conducted a cohort study assessing employment, occupational position, and income in 1469 adults with NF1 and their 11,991 matched population comparisons at ages 30, 40 and 50 years with annual outcome information from unique Danish nationwide registries.

## Patients and methods

### NF1 and comparison cohort

We have previously established a national cohort of 2467 individuals hospitalized with or for NF1 or followed on one of two national Centers for Rare Diseases at Aarhus University Hospital or Copenhagen University Hospital, Rigshospitalet (a detailed description of the cohort is available [16]) in the period 1977 to 2013. All individuals with NF1 were matched to ten general population comparisons, randomly selected from the Danish Civil Registration System and matched on birth month and year as well as sex [17]. From this cohort, we identified 1469 individuals with NF1 living in Denmark and their 11,991 matched population comparisons, who were ≥ 30 years old at start of study. Finally, we retrieved information on migration and vital status from the Danish Civil Registration System for all cohort members.

### Employment, occupational position, and income

All inhabitants in Denmark are given a unique personal identification number at birth or upon emigration to Denmark, which enables accurate linkage among national registries [17]. By linking the personal identification number of all included individuals, we ascertained information on employment, occupation and income from nationwide administrative registers updated annually by Statistics Denmark.

Information on annual employment and disposable income was obtained from the Integrated Database for Labour Market Research (IDA Register) [18], which was available in the period 1980–2019. The register contains longitudinal information on income, employment, and establishments on an individual level. We categorized employment status on annual basis into three groups: employed, unemployed (i.e., individuals who receive unemployment benefits and not health-related social security benefits during that calendar year) and unemployed for health-related reasons (i.e., individuals who received sickness or disability benefits, social assistance or rehabilitation benefits or health-related retirement during that calendar year). If an individual was registered as employed at one point during a calendar year, then the individual was categorized as employed irrespective of any other registration of unemployment during the same calendar year. Likewise, an individual was classified as unemployed for health-related reasons if any social benefits due to health reasons were received during a calendar year. Thus, employment, unemployment, and health-related unemployment were mutually exclusive, so an individual could only be registered as either employed, unemployed, or unemployed for health-related reasons during a calendar year. Finally, we did not include individuals in the employment analyses in a calendar year during which they were enrolled as students at any teaching institutions.

Also, from the IDA Register [18], we included data on annual disposable income, which is defined as the amount of money that an individual has in his or her possession after income taxes and interests have been deducted. Based on annual disposable income, we defined low income as income below the threshold set at 60% of the median disposable income, corresponding to the at-risk-of-poverty threshold used by Eurostat [19].

We obtained information on occupational positions from the Employment Classification Module (AKM Register), which collects annual information on the most important employment activity (i.e. the activity with the highest income) since 1993 [18]. We grouped occupational positions for the period 1993–2019 into 10 major groups according to the International Standard Classification of Occupation (ISCO-08) [20]: (1) managers, (2) professionals, (3) technicians and associate professionals, (4) clerical support workers, (5) services and sales workers, (6) skilled agricultural, forestry and fishery workers, (7) craft and related trades workers, (8) plant and machine operators and assemblers, (9) elementary occupations, (10) armed forces occupations. Additionally, we grouped occupational positions according to ISCO skill level (i.e., the ability to carry out the tasks and duties of a given occupation defined by the complexity and range of tasks) with highly skilled occupations defined by ISCO group 1, 2 or 3 [20].

### Highest educational attainment

We have previously reported that individuals with NF1 are less likely to achieve a medium or long education compared to the background population [9]. Thus, we linked our study population to the Danish Education Registry, which includes information on all educational programs in Denmark since 1981. The Registry ascertains the highest level of completed education on a yearly basis [21]. We included information on highest attained education before age 30 years categorized as short (≤ 9 years of education), medium (10–12 years), and long (> 12 years) education.

### Hospital contacts for somatic and psychiatric disease

We included information on somatic and psychiatric disease before age 30 years to assess their association with employment status, occupational position, and disposable income at age 30 years. From the Danish Cancer Registry [22], we retrieved all primary cancer diagnoses. Information on hospitalizations for somatic disease other than cancer was obtained from the Danish National Patient Registry [23] and categorized into six groups on a yes/no level: benign tumors, neurologic disease, gastrointestinal disease, diseases of bones, joints and soft tissue and other somatic disease. Finally, we included information on psychiatric hospital contacts from the Danish Psychiatric Central Research Register [24]. The register was established in 1969 and includes information on all patients treated at psychiatric departments in Denmark, supplemented with outpatient and emergency room contacts since 1995. Information on psychiatric diagnoses was included as any/no hospital contacts for psychiatric disease (a list of all included diagnoses has previously been published [5]).

### Statistical analyses

We assessed employment, health-related unemployment, and other unemployment as well as disposable income from age 30 years using the study cohorts of 1469 individuals with NF1 and 11,991 population comparisons. The study population was followed until date of death, emigration, or end of study period (31 December 2019), whichever occurred first. Only individuals surviving to each age were compared.

Employment, health-related unemployment, other employment, and low disposable income were defined as dichotomous outcomes. We plotted the annual prevalence of individuals in each group between ages 30 and 50 years stratified by sex. Unconditional logistic regression models were used to estimate odds ratios and 95% confidence intervals (CIs) for employment, health-related unemployment, other employment, and low disposable income adjusting for calendar period using linear splines, highest attained education, and sex. In all analyses, individuals with and without NF1 were compared at age 30, 40, and 50 years. The analyses were conducted using the whole study population alive at each age. We also conducted logistic regression analyses of employment, unemployment, and health-related unemployment at age 30 years for specific somatic disease groups (cancer, benign tumors, diseases of nervous system and sense organs, diseases of digestive organs, and diseases of bone, joints, and soft tissue) and any psychiatric disease adjusting for calendar period using linear splines, highest attained education, and sex.

We plotted the median annual disposable income between ages 30 to 50 years. We used linear regression to assess the disposable income level in individuals with NF1 compared to the general population for different birth cohorts (1929–1940, 1941–1950, 1951–1960, 1961–1970, 1971–1980, and 1981–1988). We also assessed difference in occupational position at age 30 years using the 10 major ISCO groups in a logistic regression model. Employed cohort members with missing information on occupation were included as a missing group. Finally, we estimated the odds for obtaining a highly skilled occupation (defined as ISCO group 1, 2 and 3) at ages 30, 40 and 50 years using a logistic regression model. These analyses were adjusted for calendar year using linear splines, highest attained education, and sex. We performed the analyses using R version 4.1.0 with the spline packages [25].

## Results

Characteristics of the included 1469 individuals with NF1 and 11,991 population comparisons are seen in Table 1. The median attained age at the end of follow-up was 53.5 years in individuals with NF1 and 57.7 years for the population comparisons. A higher percentage of the individuals with NF1 than the population comparisons had been hospitalized with somatic disorders before the age of 30 years, or registered with a psychiatric hospital contact. In addition, more individuals with NF1 had a short education than the comparisons.

**Table 1:** Characteristics of 1469 adults with NF1 and 11,991 population comparisons

	Adults with NF1 (N = 1469)	Population comparisons (N = 11,991)
	No. (%)	No. (%)
Sex		
Men	706 (48)	5764 (48)
Women	763 (52)	6227 (52)
Birth year		
1929–1940	203 (14)	1743 (14)
1941–1950	271 (18)	1398 (20)
1951–1960	250 (17)	2164 (18)
1961–1970	290 (20)	2452 (20)
1971–1980	273 (19)	2044 (17)
1981–1988	182 (12)	1190 (10)
Median age in years at end of follow-up (range)	53.5 (30.2–89.1)	57.7 (30.2–89.1)
Median age in years at first hospitalization for or with NF1 (range)	35.3 (0.0–79.3)	
Somatic hospital contacts (< 30 years)		
Cancer	74 (7)	34 (0)
Benign tumors	212 (21)	259 (3)
Neurologic disease	214 (21)	727 (9)
Gastrointestinal disease	211 (21)	1131 (14)
Diseases of bones, joints, and soft tissue	226 (23)	1113 (14)
Psychiatric hospital contacts (< 30 years)		
Any	76 (8)	406 (5)
Highest educational level (< 30 years)		
Short	664 (45)	3587 (30)
Medium	513 (35)	5458 (46)
Long	201 (14)	2460 (21)
Unknown	91 (6)	486 (4)

### Employment, unemployment, and health-related unemployment

**Fig. 1:**
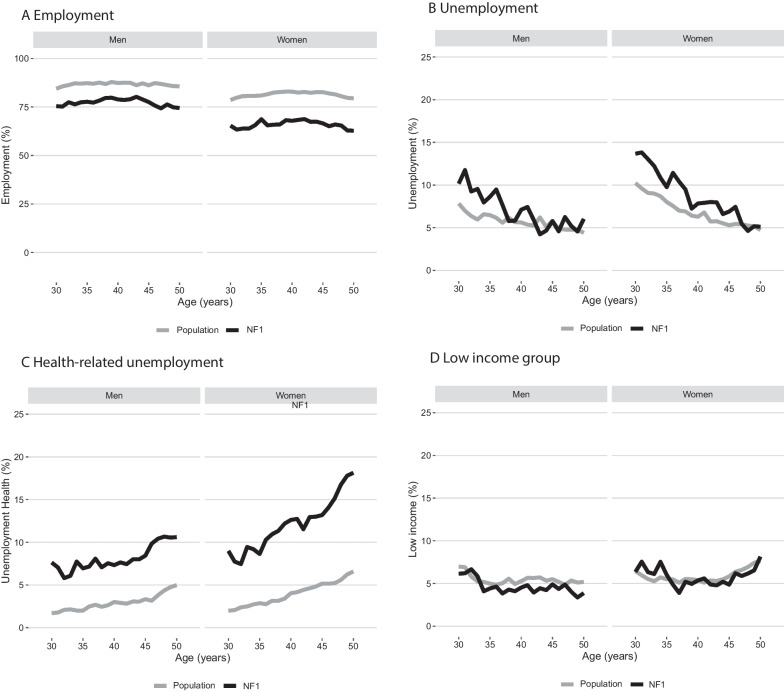
Employment (**A**), unemployment (**B**), health-related unemployment (**C**) and low income (**D**) by ages 30 to 50 years for adults with NF1 and population comparisons

Figure 1A–C show the annual proportions of employment, unemployment, and health-related unemployment at ages 30 to 50 years. At all ages, individuals with NF1 were less often employed than the comparisons, with the lowest employment rate observed in women with NF1 aged 50 years (63%; female comparisons: 79%) (Fig. 1A). The annual proportion of individuals being unemployed was similar to the comparison cohort; however, individuals with NF1 were more often unemployed due to reasons related to health. At age 50 years, 18% of women with NF1 were health-related unemployed compared to 7% of the female comparisons.

We found that individuals with NF1 had a lower OR for employment than the comparison cohort, both at age 30 (OR 0.71, 95% CI 0.61–0.83), 40 (OR 0.58, 95% CI 0.49–0.68), and 50 (0.53, 95% CI 0.45–0.62) years (Table 2). We did not find any increased OR for unemployment. At age 30 years, individuals with NF1 had an increased OR for health-related unemployment of 2.94 (95% CI 2.16–3.96), which was also increased at age 40 and 50 years. The ORs for employment were slightly lower in women with NF1 than in men with NF1 when compared with women and men from the general population, respectively. The ORs for health-related unemployment were slightly higher in women with NF1 (Additional file 1: Table S1).

**Table 2:** Odds for employment, unemployment, health-related unemployment and low income at age 30, 40 and 50 years

	Adults with NF1	Population comparisons	OR^b^ (95% CI)
	n/N^a^ (%)	n/N^a^ (%)	
Employment			
At 30 years	705/1006 (70)	6514/ 8010 (81)	0.71 (0.61–0.83)
At 40 years	721/987 (73)	7377/8680 (85)	0.58 (0.49–0.68)
At 50 years	579/845 (69)	6528/7920 (82)	0.53 (0.45–0.62)
Unemployment			
At 30 years	121/1006 (12)	729/8010 (9)	1.08 (0.87–1.33)
At 40 years	74/987 (8)	518/8680 (6)	1.07 (0.82–1.37)
At 50 years	47/845 (6)	362/7920 (5)	1.10 (0.80–1.50)
Health-related unemployment			
At 30 years	84/1006 (8)	148/8010 (2)	2.94 (2.16–3.96)
At 40 years	100/987 (10)	308/8680 (4)	2.45 (1.89–3.16)
At 50 years	122/845 (14)	460/7920 (20)	2.52 (1.99–3.18)
Low income			
At 30 years	63/1006 (6)	536/8009 (7)	0.81 (0.61–1.06)
At 40 years	49/987 (5)	463/8680 (5)	0.86 (0.62–1.15)
At 50 years	51/844 (6)	513/7920 (7)	0.82 (0.59–1.10)

^a^n = number of persons with an event (employment, unemployment, health-related unemployment, low income); N = total population a live at age 30, 40 or 50 years

^b^Adjusted for calendar year (linear splines), highest attained education and sex

We assessed previous somatic hospitalizations for cancer, benign tumors, neurological disease, gastrointestinal disease and diseases of bones, joints and soft tissues and psychiatric contacts as risk factors for employment status at age 30 years (Table 3). Individuals with NF1 who had somatic hospitalizations before age 30 years had a higher OR for being unemployed for health-related reasons than population comparisons who also had somatic hospitalizations. Due to too few cancer diagnoses in the comparison cohort, we were unable to assess cancer as a risk factor for health-related unemployment. No association was found between any psychiatric hospital contact and the different outcomes.

**Table 3:** Associations of somatic and psychiatric disease with employment, unemployment and health-related unemployment at age 30 years

	Adults with NF1	Population comparisons	OR^b^ (95% CI)
	n/N^a^ (%)	n/N^a^ (%)	
Employment at age 30 years			
Somatic hospitalization			
Cancer	44/74 (60)	27/34 (79)	0.57 (0.19–1.57)
Benign tumors	147/212 (69)	213/259 (82)	0.67 (0.42–1.08)
Neurologic disease	117/214 (55)	543/727 (75)	0.57 (0.40–0.80)
Gastrointestinal disease	132/211 (63)	858/1131 (76)	0.73 (0.52–1.02)
Diseases of bones, joints and soft tissue	146/226 (65)	856/1113 (77)	0.68 (0.50–0.95)
Psychiatric hospital contacts			
Any	32/76 (42)	203/406 (50)	1.14 (0.66–1.76)
Unemployment at age 30 years			
Somatic hospitalization			
Cancer	7/74 (10)	NA^c^	NA^c^
Benign tumors	24/212 (11)	21/259 (8)	1.17 (0.60–2.29)
Neurologic disease	28/214 (13)	79/727 (11)	0.98 (0.59–1.57)
Gastrointestinal disease	26/211 (12)	121/1131 (11)	0.94 (0.58–1.48)
Diseases of bones, joints and soft tissue	30/226 (13)	111/1113 (10)	1.11 (0.70–1.72)
Psychiatric hospital contacts			
Any	15/76 (20)	79/406 (20)	0.70 (0.36–1.30)
Health-related unemployment at age 30 years			
Somatic hospitalization			
Cancer	15/74 (20)	NA^c^	NA^c^
Benign tumors	31/212 (14)	6/259 (2)	3.84 (1.60–10.7)
Neurologic disease	50/214 (23)	47/727 (6)	2.88 (1.79–4.64)
Gastrointestinal disease	34/211 (16)	47/1131 (4)	2.67 (1.59–4.43)
Diseases of bones, joints and soft tissue	31/226 (14)	55/1113 (5)	2.19 (1.33–3.58)
Psychiatric hospital contacts			
Any	23/76 (30)	77/406 (19)	1.36 (0.75–2.43)

^a^n = number persons with a hospitalization in a specific diagnostic group with an event (employment, unemployment, health-related unemployment, low income; N = total population a live at age 30 with a hospitalization in a specific diagnostic group

^b^Adjusted for calendar year (linear splines), highest attained education and sex

^c^OR cannot be estimated due to few numbers of events in the comparison group

### Occupational position

Individuals with NF1 had lower ORs for being in high skilled occupation, including managers, professionals, and technicians and associate professionals at age 30 years compared with individuals without NF1 (Table 4); however, the OR was only significantly decreased for professionals (OR 0.60, 95% CI 0.41–0.87). When we combined the high skilled occupations, individuals with NF1 had significantly lower ORs for being in a high skilled occupation both at age 30, 40 and 50 years (Table 4).

**Table 4:** Occupational positions at different ages

	Adults with NF1	Population comparisons	OR^b^ (95% CI)
	n/N^a^ (%)	n/N^a^ (%)	
Occupational position at age 30 years			
ISCO group 1) Managers	9/705 (1)	126/6514 (2)	0.67 (0.31–1.26)
ISCO group 2) Professionals	48/705 (7)	827/6514 (13)	0.60 (0.41–0.87)
ISCO group 3) Technicians and associate professionals	59/705 (8)	806/6514 (12)	0.79 (0.59–1.06)
ISCO group 4) Clerical support workers	48/705 (7)	510/6514 (8)	0.88 (0.64–1.20)
ISCO group 5) Service and sales workers	108/705 (15)	653/6514 (10)	1.45 (1.14–1.84)
ISCO group 6) Skilled agricultural, forestry and fishery workers	4/705 (1)	64/6514 (1)	0.58 (0.17–1.42)
ISCO group 7) Craft and related trades workers	45/705 (6)	467/6514 (7)	0.89 (0.63–1.23)
ISCO group 8) Plant and machine operators, and assemblers	44/705 (6)	255/6514 (4)	1.24 (0.87–1.74)
ISCO group 9) Elementary Occupations	63/705 (9)	327/6514 (5)	1.39 (1.02–1.86)
ISCO group 10) Armed forces occupation	3/705 (0)	43/6514 (1)	0.60 (0.14–1.67)
High-skilled occupation			
At 30 years	116/705 (17)	1759/6514 (27)	0.52 (0.38–0.69)
At 40 years	149/721 (21)	2215/7377 (30)	0.57 (0.44–0.73)
At 50 years	138/579 (24)	2098/6528 (32)	0.67 (0.51–0.86)

^a^n = number of persons with the occupation; N = total population a live at age 30, 40 or 50 years

^b^Adjusted for calendar year (linear splines), highest attained education and sex

### Income

**Fig. 2:**
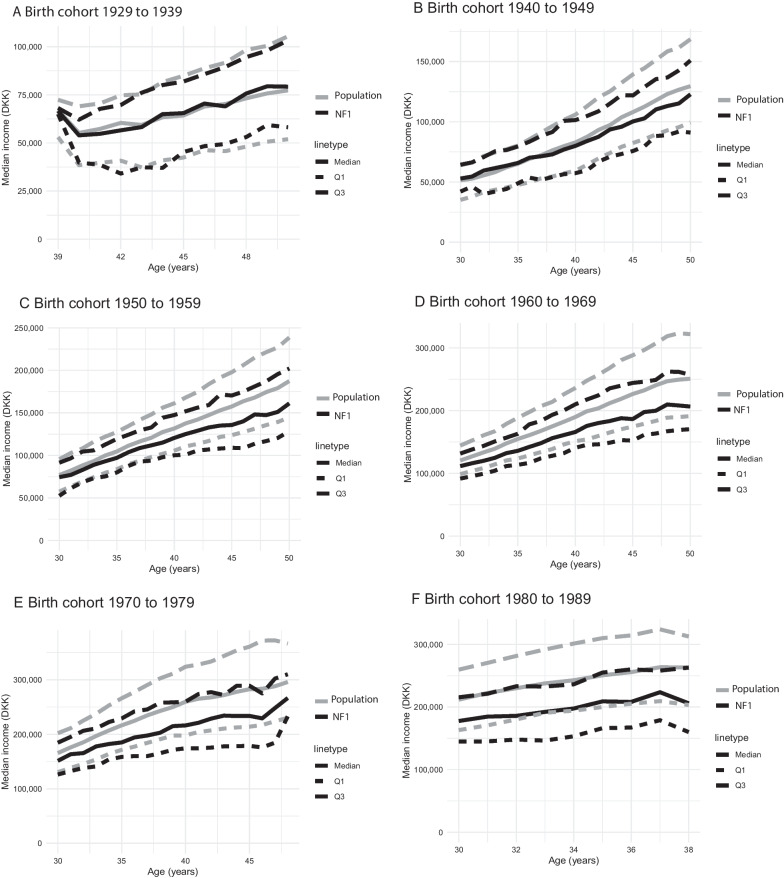
Median income with 25 (Q1) and 75 (Q3) percentile by age for different birth cohorts

Figure 2 shows the median, 25th and 75th percentile annual disposable income by age for different birth cohorts. The difference in disposable income between individuals with NF1 and the comparison group increased for the youngest birth cohorts, where the median disposable income for those with NF1 was almost similar to the 25th percentile of the comparison group. The results from the linear regression revealed that adults with NF1 had a slightly lower disposable income than the population comparisons, with a 14% reduction observed among the youngest birth cohort (Additional file 2: Table S2). However, individuals with NF1 did not have increased ORs for being in the low disposable income group either at age 30, 40 or 50 years (Table 2).

## Discussion

Information on employment, occupational, and income from high-quality registries and long follow-up enabled us to assess annual employment status, occupational positions, and disposable income in both early and late adulthood among 1469 individuals with NF1. We found that a considerable number of individuals with NF1 were not employed, which was mainly due to health-related reasons. Medical diseases were associated with increased health-related unemployment. The disposable income was decreased among adults with NF1, with an income of up to 14% lower of the comparison cohort in the youngest birth cohort. Finally, we found that individuals with NF1 were less likely to be employed in high skilled occupations.

According to our knowledge, this is the first study to report sex-specific employment status in individuals with NF1 using a nation-wide and population-based study design. At all ages, the employment rates were lower in individuals with NF1 than in population comparisons, with the lowest rate observed among women with NF1. Several factors are associated with employment status, including educational attainment, where higher educational attainment has been found to decrease long-term unemployment [26]. Two large population-based cohort studies in Denmark and Finland have reported reduced educational achievements in individuals with NF1 [9, 10], with a tendency of vocational over academic education in individuals with NF1 [10]. Using the same Danish NF1 cohort, we have also shown that children with NF1 have significantly lower school grades than their NF1-free peers when finalizing mandatory school, and the largest difference was seen for girls with NF1 [27]. The lower grades, shorter educational level, and higher odds for less skilled occupation might reduce the number of potential jobs for women with NF1 and explain why fewer women than men with NF1 are employed. Our findings of lower employment rates in adults with NF1 are in line with the results of a recently published Finnish study. This study reported a reduced number of working days per year as well as higher rates of unemployment and sickness absence among 742 individuals with NF1 compared to a matched control population [14]. However, both the Finnish study and our research employed a hospital-based design to identify individuals with NF1, which needs to be considered when interpreting the results.

Individuals with NF1 have a higher psychiatric disease burden, mainly due to increased risks for ADHD and autism [5]. In addition, intellectual problems, including a lower intelligence quotient, are a recognized feature of NF1 [28] and have a negative impact on school performance [29]. However, we did not have enough power to assess specific psychiatric disorders and their association with employment status. Furthermore, the high somatic disease burden reported in individuals with NF1 has also been found to be associated with an increased number of sickness absence in Finnish adults with NF1 [14]. In 2019, Fjermestad examined health complaints and work experiences in 142 Norwegian adults with NF1 using a control group and normative data. More adults with NF1 reported work as physically exhausting and had more subjective health complaints. They also more often reported work bullying [30]. This is in line with our results of an increased OR for health-related unemployment at all ages.

A consequence of lower employment rates and being in occupational groups with low salaries is a reduced income. We also found that individuals with NF1 had a reduced disposable income of approximately 10%. Recently, a Finnish population-based study reported that short education, increased morbidity, and reduced labor market participation partly explained a lower income in Finnish individuals with NF1 [14]. Denmark and Finland have similar welfare and health-care systems characterized by high taxes, universal and tax-funded health-care systems, free education, and high social security benefits compared to other countries. Thus, the disposable income is probably even lower in adults with NF1 living outside the Nordic countries. We found that the difference in disposable income between individuals with NF1 and the population comparisons increased for the younger birth cohorts, which is in line with the trend of an increase in income equality reported in the general population of the Nordic countries [31].

### Strengths and limitations

The strengths of the study include the population-based design with information on employment, income, and occupation from high-quality nationwide registries, irrespective of NF1 status, which reduced the risk of information bias from self-reported data and limited loss to follow-up. We were also able to compare the employment history in individuals with NF1 with that of randomly selected population comparisons to represent the Danish background population. The NF1 patients identified in the two Centers for Rare Diseases fulfilled the US National Institutes of Health (NIH) criteria for NF1 [32] or had their diagnosis confirmed by molecular genetic testing. For the patients identified in the Danish Patients Register, we excluded those with clinical and anatomic features of NF2, but some misclassification of the NF1 diagnosis cannot be ruled out. As the individuals in the NF1 cohort had all been hospitalized with or for NF1 or are followed in the Centers for Rare Diseases, we might have missed individuals with NF1 who are less affected by their NF1. However, all Danish inhabitants are encouraged to seek follow-up care at one of the two Centers for Rare Diseases, regardless of the severity of their disease. Follow-up services are provided free of charge, which reduces the risk of selection of participants due to financial constraints. In addition, the process of diagnosing NF1 will result in at least one registration in the RAREDIS Database, even if the patient declines further follow-up at the Center for Rare Diseases. Despite these initiatives, some individuals with mild symptoms or asymptomatic relatives may choose not to undergo clinical investigation for NF1, and consequently, they will not be included in our NF1 cohort. The employment rates and income equality might have been improved if we were able to include study participants not affiliated with the two Centers for Rare Diseases. Thus, our results should not be generalized to adults with NF1 who are only in contact with the primary health care system. Furthermore, we included some who received both benefits within the same calendar year in our definition of unemployment and health-related employment. However, this potential misclassification is expected to be similar for adults with and without NF1, thus biasing towards the null. Another limitation of the study is the inclusion of psychiatric hospital contacts using the Danish Psychiatric Central Research Register, which only holds information on outpatient contacts since 1995. Most patients with depression and anxiety as well as attention deficit/hyperactivity disorder and autism spectrum disorders are not hospitalized. Consequently, we were not able to assess the association between specific psychiatric disorders and employment status. Finally, the study was conducted in Denmark, where social security benefits are higher than in other countries. Thus, the gap in disposable income between adults with and without NF1 might be higher in countries outside Denmark and other Nordic countries.

## Conclusion

In conclusion, we found that individuals with NF1 are less employed than the background population, which was mainly due to health-related reasons. Furthermore, this vulnerable patient group is also less likely to have a high-skilled occupation. The disposable income was reduced in adults with NF1, with largest difference in income observed for the younger birth cohorts. However, adults with NF1 were not at increased risk for being in the lowest disposable income group. Employment and work experience is beneficial for health and well-being, also in disabled persons [33], thus it is important that the health care system, whether privately or publicly funded, will prioritize rehabilitation of patients with NF1 to optimize and ensure their attachment to the labor market. However, also educators of young people with NF1 might use our findings to inform career counselling during the formative years. In general, anticipation guidance for employment should be part of the management of NF1 families.

### Supplementary Information

**Additional file 1: Table s1**. Odds for employment, unemployment, health-related unemployment and low income in adults with NF1 and population comparisons.

**Additional file 2: Table s2**. Income of adults with NF1 compared with population comparisons by birth cohort.

## Data Availability

The data that support the findings of this study were accessed remotely on a secure platform at Statistics Denmark. Data are available from Statistics Denmark, but restrictions apply to the availability of these data, which were used under license for the current study, and so not publicly available. Researchers, who fulfilled the Danish legal requirements for access to personal sensitive data, can apply for access. Please contact daily coordinator of the Danish NF1 Research Program, Line Kenborg (kenborg@cancer.dk), or Principal Investigator, Jeanette Falck Winther (jeanette@cancer.dk) for further questions about data access.

## Abbreviations

ADHD: Attention-deficit/hyperactivity disorder
ASD: Autism spectrum disorder
CI: Confidence interval
ISCO: International Classification of Occupation
NF1: Neurofibromatosis 1
NIH: National Institutes of Health
OR: Odds ratio
