# Supplementary material for: Employment, occupation, and income in adults with neurofibromatosis 1 in Denmark: a population- and register-based cohort study
Source: Orphanet J Rare Dis. 2023 Nov 6;18:346. doi: 10.1186/s13023-023-02965-2 (PMC10629102; doi:10.1186/s13023-023-02965-2)
Supplement: Supplementary file 1 — Additional file 1: Table s1. Odds for employment, unemployment, health-related unemployment and low income in adults with NF1 and population comparisons. [file 13023_2023_2965_MOESM1_ESM.docx]

**Supplementary Table 1. Odds for employment, unemployment, health-related unemployment and low income in adults with NF1 and population comparisons at age 30, 40 and 50 years stratified by sex**

|  | Women | | | Men | | |
| --- | --- | --- | --- | --- | --- | --- |
|  | Women with NF1 | NF1 free women | OR^c^ (95% CI) | Men with NF1 | NF1 free men | OR^c^ (95% CI) |
|  | n/N^a^ (%) | n/N^a^ (%) |  | n/N^a^ (%) | n/N^a^ (%) |  |
| **Employment** |  |  |  |  |  |  |
| At 30 years | 349/534 (65) | 3335 / 4243 (79) | 0.67 (0.55–0.82) | 356/472 (75) | 3179/3767 (84) | 0.76 (0.60–0.98) |
| At 40 years | 355/523 (68) | 3800/4584 (83) | 0.52 (0.43–0.65) | 366/464 (79) | 3577/4096 (87) | 0.65 (0.50–0.83) |
| At 50 years | 270/430 (63) | 3259 / 4102 (79) | 0.48 (0.39–0.60) | 309/415 (75) | 3269/3818 (86) | 0.56 (0.44–0.73) |
| **Unemployment** |  |  |  |  |  |  |
| At 30 years | 73/534 (14) | 434/4243 (10) | 1.11 (0.84–1.45) | 48/472 (10) | 295/3767 (8) | 1.04 (0.74–1.44) |
| At 40 years | 41/523 (8) | 288/4584 (6) | 1.03 (0.72–1.45) | 33/464 (7) | 230/4096 (6) | 1.11 (0.74–1.60) |
| At 50 years | 22/430 (5) | 194/4102 (5) | 0.98 (0.61–1.52) | 25/415 (6) | 168/3818 (4) | 1.23 (0.77–1.86) |
| **Health-related unemployment** |  |  |  |  |  |  |
| At 30 years | 48/534 (9) | 84 / 4243 (2) | 2.97 (1.98–4.43) | 36/472 (8) | 64/3767 (2) | 2.84 (1.78–4.49) |
| At 40 years | 66/523 (13) | 185/4584 (4) | 2.77 (1.99–3.81) | 34/464 (7) | 123/4096 (3) | 2.03 (1.32–3.07) |
| At 50 years | 78/430 (18) | 270 / 4102 (7) | 3.01 (2.22–4.05) | 44/415 (11) | 190/3818 (5) | 1.98 (1.35–2.85) |
| **Low income** |  |  |  |  |  |  |
| At 30 years | 34/534 (6) | 273/4243 (6) | 0.84 (0.57–1.22) | 29/472 (6) | 263/3766 (7) | 0.78 (0.51–1.16) |
| At 40 years | 28/523 (5) | 247/4584 (5) | 0.95 (0.62–1.42) | 21/464 (5) | 216/4096 (5) | 0.77 (0.47–1.20) |
| At 50 years | 35/430 (8) | 315/4102 (8) | 0.98 (0.66–1.43) | 16/414 (4) | 198/3818 (5) | 0.65 (0.37–1.07) |

^a^ n=number of persons with an event (employment, unemployment, health-related unemployment, low income); N=total population a live at age 30, 40 or 50 years

^b^ Adjusted for calendar year (linear splines), highest attained education and sex
