# Supplementary material for: Employment, occupation, and income in adults with neurofibromatosis 1 in Denmark: a population- and register-based cohort study
Source: Orphanet J Rare Dis. 2023 Nov 6;18:346. doi: 10.1186/s13023-023-02965-2 (PMC10629102; doi:10.1186/s13023-023-02965-2)
Supplement: Supplementary file 2 — Additional file 2: Table s2. Income of adults with NF1 compared with population comparisons by birth cohort. [file 13023_2023_2965_MOESM2_ESM.docx]

**Supplementary Table 2. Income from age 30 to 50 years of individuals with NF1 compared with population comparisons by birth cohort^a^**

|  | Coefficient (95% CI) |
| --- | --- |
| **Birth cohort** |  |
| 1929–1940 | 0.94 (0.83–1.06) |
| 1941–1950 | 0.92 (0.88–0.96) |
| 1951–1960 | 0.90 (0.88–0.93) |
| 1961–1970 | 0.87 (0.85–0.88) |
| 1971–1980 | 0.90 (0.88–0.91) |
| 1981–1988 | 0.86 (0.82–0.89) |

^a^ Estimated by linear regression on log income adjusted for sex
